# A Case of Recalcitrant Plantar Warts Associated with Statin Use

**DOI:** 10.1155/2015/320620

**Published:** 2015-02-18

**Authors:** Aaron G. Wernham, Shireen S. Velangi

**Affiliations:** Department of Dermatology, Sandwell and West Birmingham NHS Trust, Birmingham B18 7QH, UK

## Abstract

*Background*. Plantar warts are a common presenting skin complaint caused by the human papillomavirus. 1st line therapies include cryotherapy and topical salicylic acid. Where there is resistance to these treatments, consideration is made for 2nd line therapies, including intralesional bleomycin, imiquimod, 5-fluorouracil, and photodynamic therapy. We present a case of bilateral persistent plantar warts, resistant to treatment with repeated cryotherapy and topical salicylic acid over a 6-year period. Following a patient initiated decision to discontinue their statin medication, we observed rapid clearance of plantar warts without change to standard therapy or their environment. This case correlates with emerging literature demonstrating a link between statin medication and proliferation of HPV through increased levels of FOXP3+ regulatory T cells.

## 1. Introduction

Cutaneous warts represent a common cutaneous manifestation of the human papillomavirus (HPV), most frequently occurring on the hands and feet. Initial skin treatment may involve repeated course two-cycle cryotherapy and topical salicylic acid [1]. A subgroup of patients are resistant to these treatments and require second line management. Treatments shown to be efficacious as second line therapies include intralesional bleomycin, imiquimod, 5-fluorouracil, and photodynamic therapy [1]. We report a case of persistent bilateral plantar warts, resolving following discontinuation of statin therapy. This case correlates with emerging literature demonstrating a link between statin medication and proliferation of HPV through increased levels of FOXP3+ regulatory T cells.

## 2. Case Report

A 67-year-old Caucasian gentleman was referred to our department with a six-year history of bilateral persistent plantar warts. Initial treatment prior to specialist opinion involved several courses of cryotherapy and a topical combination of salicylic acid 16.7% and lactic acid 16.7%. This was found to be minimally effective.

On review, there was no history of atopy and no medical predisposition for developing recalcitrant viral warts. The patient's medications included clopidogrel, perindopril, and simvastatin for secondary prevention of cardiovascular disease and quinine sulphate for leg cramps. On examination, there were several warts to the sole of each foot but no abnormal findings on other skin, hair, or nail examination. In view of the resistance to first line therapies, a referral was made to trial photodynamic therapy (PDT).

The patient returned to dermatology for review 6 months later. Examination of his feet revealed a dramatic clearance of viral warts with no active lesions. This occurred prior to his appointment for photodynamic therapy, which had, therefore, been cancelled. Discussion with the patient revealed that he had discontinued his statin medication, correlating with subsequent improvement. There were no other changes to his medical history, environment, or treatment that could be identified.

## 3. Discussion

In view of the remarkable improvement following statin discontinuation, we reviewed current literature for previously documented effects of statins on the growth of HPV.

Statins are commonly prescribed as an effective lipid lowering agent, which work by inhibiting the enzyme HMG-CoA reductase. Although cholesterol levels are reduced to similar levels compared with other lipid lowering agents, they have been shown to be more effective in reducing cardiovascular outcomes. This is thought to be due to an additional effect on immune modification [2, 3]. One of these effects is to increase FOXP3+ regulatory T cells (Treg), which play an immunosuppressive role in the body as a surveillance measure against development of autoimmunity [4].

Two authors have looked at the association of Treg cells with HPV related conditions. Molling et al. [5] investigated the role of Treg cells in cervical intraepithelial neoplasia (CIN) and found increased frequencies of Treg cells in patients with persistent HPV-16 infection. The Treg cells were considered to be causing local immunosuppression, thereby facilitating HPV growth. Cao et al. [6] investigated the role of Treg cells in HPV related genital condylomata accuminata and found higher levels in larger warts, also suggesting that they were facilitating HPV proliferation.

Mausner-Fainberg et al. [7] subsequently reviewed the effect of statins on Treg cells and found an association with increased numbers of Treg cells. This was shown to occur through induction of the transcription factor forkhead P3. It led to the hypothesis that statins have potential to increase the risk of uncontrolled HPV growth [8].

A further mechanism linking statins to HPV proliferation has been proposed by Feingold [9]. They suggest that statins inhibit cholesterol synthesis in keratinocytes, which may lead to impairment of barrier function and enable HPV to proliferate within the skin.

We report a case demonstrating resolution of recalcitrant plantar warts following statin discontinuation. This may be caused by increased Treg cell production, leading to local immunosuppression at the sites of viral warts. Further cases are required to establish the importance of this link, particularly in patients where treatment resistance exists.

## References

[1] Kwok C. S., Gibbs S., Bennett C., Holland R., Abbott R. (2012). Topical treatments for cutaneous warts. *Cochrane Database of Systematic Reviews*.

[2] Maron D. J., Fazio S., Linton M. F. (2000). Current perspectives on statins. *Circulation*.

[3] Mason J. C. (2003). Statins and their role in vascular protection. *Clinical Science*.

[4] O'Garra A., Vieira P. (2004). Regulatory T cells and mechanisms of immune system control. *Nature Medicine*.

[5] Molling J. W., de Gruijl T. D., Glim J. (2007). CD4^+^CD25^hi^ regulatory T-cell frequency correlates with persistence of human papillomavirus type 16 and T helper cell responses in patients with cervical intraepithelial neoplasia. *International Journal of Cancer*.

[6] Cao Y., Zhao J., Lei Z. (2008). Local accumulation of FOXP3^+^ regulatory T cells: evidence for an immune evasion mechanism in patients with large condylomata acuminata. *The Journal of Immunology*.

[7] Mausner-Fainberg K., Luboshits G., Mor A. (2008). The effect of HMG-CoA reductase inhibitors on naturally occurring CD4^+^CD25^+^ T cells. *Atherosclerosis*.

[8] Goldstein M. R., Mascitelli L., Pezzetta F. (2008). Comment on ‘Local accumulation of FOXP3+ regulatory T cells: evidence for an immune evasion mechanism in patients with large condylomata acuminata’. *The Journal of Immunology*.

[9] Feingold K. R. (2007). Thematic review series: skin lipids. The role of epidermal lipids in cutaneous permeability barrier homeostasis. *Journal of Lipid Research*.

